# Interobserver Reproducibility of the Visual Echocardiographic Scoring System for Left Ventricular Filling Pressure

**DOI:** 10.7759/cureus.103693

**Published:** 2026-02-16

**Authors:** Yuka Uruma, Michito Murayama, Wahei Uemura, Namiko Sakai, Midori Shimomura, Kaori Nozaki, Yasuyuki Kunieda

**Affiliations:** 1 Department of Internal Medicine, Wakkanai City Hospital, Wakkanai, JPN; 2 Department of Medical Laboratory Sciences, Faculty of Health Sciences, Hokkaido University, Sapporo, JPN; 3 Diagnostic Center for Sonography, Hokkaido University Hospital, Sapporo, JPN; 4 Department of General Medicine, Rishiri Island National Health Insurance Central Hospital, Rishiri Island, JPN; 5 Clinical Laboratory Section, Wakkanai City Hospital, Wakkanai, JPN

**Keywords:** echocardiography, heart failure, interobserver agreement, left ventricular filling pressure, reproducibility, vmt score

## Abstract

Background

The visually assessed time difference between mitral and tricuspid valve opening (VMT) scoring is a simple B-mode echocardiographic method for estimating left ventricular filling pressure (LVFP). This study aimed to evaluate the interobserver reproducibility of VMT scoring between a resident doctor using a handheld ultrasound device and experienced sonographers using a stationary system.

Methodology

In this prospective observational study, 27 inpatients underwent two echocardiographic examinations on the same day: a handheld ultrasound performed by a resident doctor and standard echocardiography performed by cardiac sonographers. Both observers independently assessed VMT scores (0-3) based on the time sequence of atrioventricular valve opening and inferior vena cava findings. Interobserver agreement was evaluated using weighted kappa statistics.

Results

Among the 27 patients (mean age = 76 ± 12 years; 56% female), 11 (41%) had a VMT score ≥2, indicating elevated LVFP. The weighted kappa value for interobserver agreement was 0.97, demonstrating almost-perfect concordance regardless of operator experience or device type.

Conclusions

VMT scoring appears to be a practical and reliable method for LVFP evaluation in diverse clinical settings, particularly those with limited access to expert echocardiography.

## Introduction

Heart failure (HF) is a leading cause of hospitalization and requires effective management [1]. Echocardiography plays a vital role in the assessment of patients with HF. Left ventricular (LV) filling pressure (LVFP) is an important parameter in echocardiographic evaluation, with an elevated LVFP being associated with poor clinical outcomes in patients with HF [2]. While the early diastolic opening of the tricuspid valve (TV) precedes that of the mitral valve (MV) in normal conditions [3,4], once LVFP is elevated, MV opening occurs early and precedes TV opening, resulting from the early crossover of the left atrium (LA) and LV pressures [3,5,6]. Reduced right ventricular (RV) relaxation due to post-capillary pulmonary hypertension also accentuates this time sequence [3,7]. Accordingly, early MV opening is considered to reflect the degree of LVFP elevation. Several recent studies have validated that the time delay of TV opening relative to MV opening reflects LVFP in patients with HF [8-11].

The visually assessed time difference between MV and TV opening, the VMT score, has emerged as a promising tool for LVFP estimation using only the B-mode in echocardiography [8-10]. Previous studies have reported that a VMT score of ≥2 was associated with adverse clinical outcomes in patients with HF, showing a wide range of LV ejection fraction [2,12]. Furthermore, the VMT score exhibited high diagnostic accuracy for patients with acute HF presenting with dyspnea, outperforming lung ultrasonography [13]. These findings indicate the potential of the VMT score to improve HF diagnosis and risk stratification.

The recent development of handheld ultrasound (US) devices has the potential to revolutionize the availability and integration of echocardiography into medical practice, particularly for noncardiology medical professionals [14]. However, whether the VMT score can be correctly assessed by beginners in echocardiography and handheld US remains unknown. Therefore, this study aimed to evaluate the interobserver reproducibility of the VMT score between a novice physician using a handheld US device and experienced cardiac sonographers using a standard echocardiography system.

This article was previously posted to the Authoria preprint server on October 6, 2025 [15].

## Materials and methods

Study population

We conducted a prospective observational study including adult patients admitted to Wakkanai City Hospital from June to July 2024. We initially enrolled 30 consecutive patients scheduled to undergo echocardiography performed by cardiac sonographers. Patients with poor echocardiographic images were excluded. Ultimately, 27 patients were included in the final analysis. This study was reviewed and approved by the Institutional Ethics Committee of Wakkanai City Hospital (approval number: R6-04; approval date: May 31, 2024).

Echocardiographic examination

**Figure 1:**
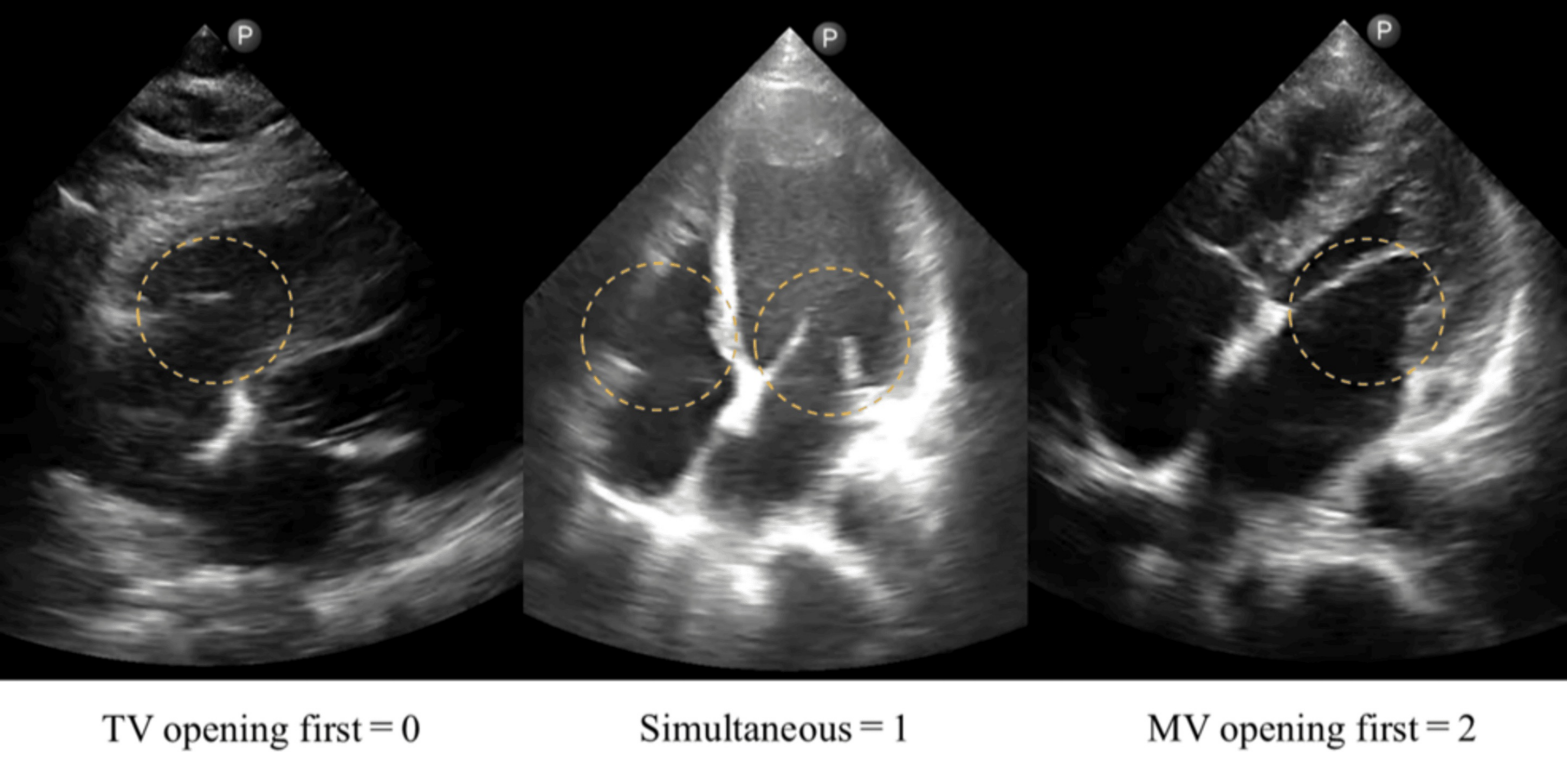
The VMT score was graded as 0 for TV opening first, 1 for simultaneous opening, and 2 for MV opening first. VMT = visually assessed time difference between mitral and tricuspid valve opening; TV = tricuspid valve; MV = mitral valve

Each patient underwent two echocardiographic examinations on the same day within five hours. The resident doctor had performed approximately 30 transthoracic echocardiographic examinations during the six months before this study. First, transthoracic echocardiography was performed by a resident doctor at the bedside using a handheld US (Lumify with S4-1 sector probe, Philips, Amsterdam, the Netherlands) on the hospital ward. Subsequently, a standard transthoracic echocardiography was performed by cardiac sonographers on the same patient in the echocardiography laboratory using a high-end stationary US (EPIQ Elite; Philips Healthcare, Andover, MA, USA) equipped with a 1.0-5.0 MHz phased-array transducer (S5-1). The frame rate was 20-28 Hz for the handheld US system and 52 ± 7 frames per second (fps) (range = 42-67 fps) for the stationary system. The resident and the sonographers were blinded to each other’s findings. Both observers independently evaluated the VMT score for each patient. The VMT score has been evaluated as a marker of elevated LVFP [8-10]. Based on the earlier opening of the MV than the TV when LVFP exceeds right atrial (RA) pressure, the scoring system consists of (i) visual assessment of the time sequence of atrioventricular valve openings and (ii) estimation of RA pressure based on inferior vena cava (IVC). To observe the time sequence of atrioventricular valve opening, apical or subcostal four-chamber images with a depth of 15-20 cm were acquired. Adequate image quality was defined as visualization of both the MV and TV in either the apical or subcostal four-chamber view. To ensure clear delineation of valve motion, the B-mode gain was adjusted to ≥50 whenever possible. The time sequence of the MV and TV opening was visually assessed using slow playback. For each examination, cine loops including at least three cardiac cycles were reviewed frame-by-frame to determine the order of valve opening, and graded as follows: 0 = TV opening first; 1 = simultaneous; 2 = MV opening first (Figure 1). When a marker of abnormal RA pressure (IVC diameter >21 mm with <50% collapse on sniffing) was detected [16], 1 point was added, and the VMT score was calculated as four grades from 0 to 3. Then, VMT ≥2 was considered to indicate an elevated LVFP.

Outcome assessment

Other physicians or surgeons confirmed the clinical diagnosis based on physical examination, laboratory data, chest X-ray, electrocardiogram findings, echocardiographic findings other than the VMT score, and the clinical course at follow-up.

Statistical analyses

Continuous variables were expressed as mean ± standard deviation. Student’s t-test was used to compare continuous variables between groups. When appropriate, categorical variables were expressed as numbers (%) and compared using the chi-square test or Fisher’s exact test. Weighted kappa statistics were used to assess interobserver agreement for VMT score grading [17]. As a 2- or 3-point difference in the VMT score between observers represents greater disagreement than a 1-point difference, the weighted kappa was calculated (assigning partial agreement for a 1-point difference) instead of a standard (unweighted) kappa. SPSS version 25 for Windows (IBM Corp., Armonk, NY, USA), R software version 4.0.3 (R Foundation for Statistical Computing, Vienna, Austria), and Excel version 2024 (BellCurve for Excel; Social Survey Research Information, Tokyo, Japan) were used for all statistical analyses. For all tests, a p-value <0.05 was considered statistically significant.

## Results

Patient characteristics

Table 1 presents the clinical characteristics of the 27 patients stratified by the VMT score. Among them, 16 (59%) had a VMT score of 0 or 1, and 11 (41%) had a score of 2 or 3. Preoperative examinations were more predominant in patients with a VMT score of ≤1. Ischemic heart disease was the most common underlying cardiac disease, and almost half of the patients had hypertension.

**Table 1:** Demographic and clinical patient characteristics. Continuous data are expressed as means ± standard deviations, whereas categorical data are presented as n (%). The p-values are from Student’s t-test or Fisher’s exact test. VMT = visually assessed time difference between the mitral valve and tricuspid valve opening; NA = not applicable

Demographics	All patients	VMT 0 or 1	VMT 2 or 3	P-value
Number, n (%)	27	16 (59)	11 (41)	NA
Age (years), mean ± SD	76 ± 12	72 ± 12	81 ± 12	0.095
Female, n (%)	15 (56)	8 (50)	7 (64)	0.696
Male, n (%)	12 (44)	8 (50)	4 (36)	0.696
Electrocardiography, n (%)				
Atrial fibrillation	9 (33)	3 (19)	6 (55)	0.097
Others	3 (11)	1 (6)	2 (18)	0.549
Cardiac disease, n (%)				
History of heart failure	5 (19)	1 (6)	4 (36)	0.125
Ischemic heart disease	6 (22)	2 (13)	4 (36)	0.187
Valvular heart disease	4 (15)	2 (13)	2 (18)	>0.999
Comorbidity, n (%)				
Hypertension	13 (48)	8 (50)	5 (45)	>0.999
Diabetes mellitus	7 (26)	4 (25)	3 (27)	>0.999
Dyslipidemia	8 (30)	5 (31)	3 (27)	>0.999
Chronic kidney disease	5 (19)	2 (13)	3 (27)	0.371
Purpose, n (%)				
Heart failure evaluation	6 (22)	2 (13)	4 (36)	0.187
Post-acute coronary syndrome	4 (15)	1 (6)	3 (27)	0.273
Preoperative exam	9 (33)	8 (50)	1 (9)	0.042
Others	8 (30)	5 (31)	3 (27)	>0.999

Reproducibility of the VMT score

Interobserver agreement analysis was conducted based on the VMT scores determined by the resident (Y.U.) and the cardiac sonographers (N.S., M.S., and K.N.) for each patient who were blinded to the clinical, hemodynamic, and other echocardiographic data. The weighted kappa value was 0.97, indicating an almost-perfect agreement in the VMT grading between the novice and the expert observers (Table 2).

**Table 2:** Reproducibility of the VMT score. VMT = visually assessed time difference between the mitral valve and tricuspid valve opening; US = ultrasound

		Sonographer with stationary US				
		0	1	2	3	Total
Resident with portable US	0	2	1	0	0	3
	1	2	9	1	0	12
	2	0	2	6	1	9
	3	0	0	1	2	3
	Total	4	12	8	3	27

## Discussion

This study found that the VMT score can be evaluated with excellent interobserver reproducibility, even when the assessments are conducted by individuals with different levels of US experience and using different echocardiography equipment. The almost-perfect agreement between the resident using a handheld device and the experienced sonographers using a standard machine highlights the robustness of VMT scoring.

**Figure 2:**
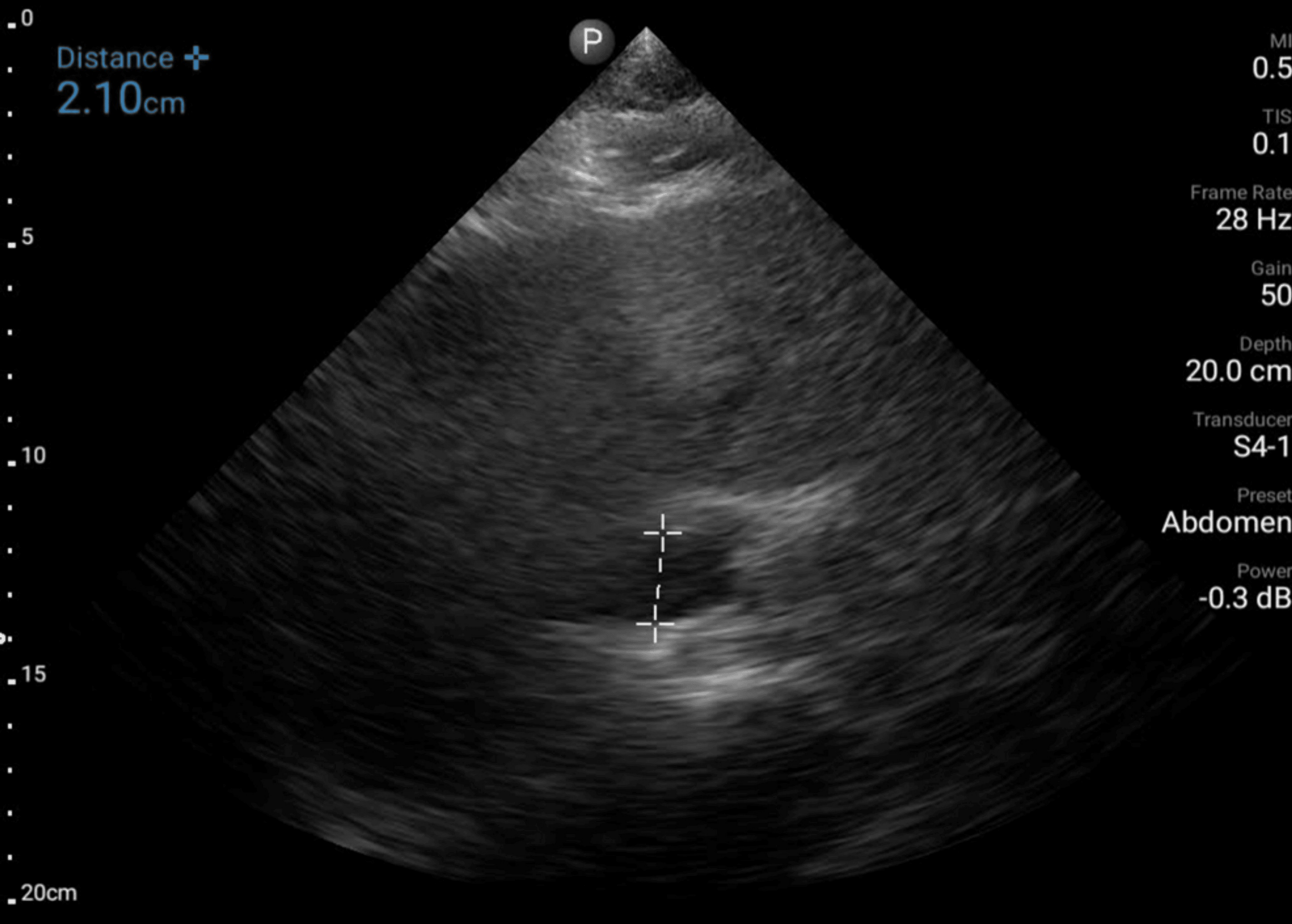
Misinterpreting the aorta instead of IVC. IVC = inferior vena cava

Although the interobserver agreement for the VMT score exhibited high reproducibility, discrepancies were observed in 3 out of 27 cases (Table 2), resulting in disagreement in terms of the presence or absence of elevated LVFP. These discrepancies were found to primarily arise from errors in the IVC measurement, where the resident doctor either measured the aorta instead of the IVC or relied on unclear echocardiographic images (Figure 2), leading to incorrect assessment of the RA pressure component. To reduce such misidentification, the use of color Doppler imaging may help differentiate venous flow in the IVC from pulsatile arterial flow in the aorta. In addition, obtaining images from a right intercostal approach or alternative subcostal windows may improve visualization of the IVC and prevent measurement errors, particularly in patients with suboptimal acoustic windows. The findings of this study indicate the potential of VMT scoring as a reliable and reproducible method for LVFP estimation, even for less-experienced practitioners. Moreover, as both handheld and stationary US can accurately measure the VMT score, the VMT score can be evaluated regardless of the type of US device. However, challenges remain, particularly in patients with suboptimal imaging conditions, such as those who are overweight or unable to assume optimal positions. In these cases, the acquisition of clear MV and TV images is difficult, which can compromise accuracy. Although apical and subcostal four-chamber views were effective for VMT score assessment, the incorporation of parasternal views or subcostal window may enhance visualization and accuracy in difficult cases. The simplicity of the VMT score, which does not require Doppler functionality, highlights its utility in resource-constrained settings and its accessibility for clinicians with varying levels of US expertise.

Recently, the American Society of Echocardiography proposed updated 2025 guidelines for LVFP assessment, introducing new algorithms that incorporate multiple indices, including Doppler parameters [18]. Although this new algorithm has been validated against invasive hemodynamic measurements [19] and its prognostic impact in daily practice has been shown [20], it requires complex parameters, such as Doppler indices and left atrial strain. The technical demands and learning curve associated with these measurements make it difficult for noncardiology medical professionals to implement the algorithm in busy primary care or emergency settings. Furthermore, atrial fibrillation (AF) is a common comorbidity whose prevalence increases with advancing age and is frequently associated with HF; for example, more than 30% of patients with HF have AF [21]. Although the 2025 guidelines provide specific algorithms for AF [18,22], these algorithms remain inherently complex and include parameters such as a body mass index (BMI) >30 kg/m². The clinical value of this BMI threshold, which is based on Western populations, remains unclear when applied to Asian populations, where obesity-related phenotypes differ. Contrarily, multicenter studies have shown that the VMT score is an effective and reliable tool even in patients with AF [10,23]. Its reliance on simple B-mode findings rather than complex hemodynamics indicates its significance as a practical imaging biomarker for HF, providing high accessibility for nonspecialists in diverse clinical settings.

Limitations

The present study has several limitations that need to be acknowledged. First, echocardiographic examinations were not performed simultaneously, and the interval between the handheld and stationary scans was up to five hours. In patients with HF, LVFP may change rapidly depending on volume status, treatment, or hemodynamic instability. Therefore, some interobserver disagreement may have been influenced by true physiological variation over time rather than observer-related error. Second, a single resident performed all the handheld scans, whereas the standard examinations were performed by multiple sonographers. While this reflects real-world practice, it could introduce variability in the method. Third, this study focused only on interobserver agreement, and intraobserver reproducibility was not examined. Future studies should assess whether VMT scoring remains consistent when repeated by the same examiner. Fourth, as the study only included patients who were admitted and scheduled for echocardiography, potential selection bias could not be excluded. Finally, the small sample size may have limited the statistical power to detect true differences, increasing the risk of a type II error, particularly when comparing individual groups shown in Table 2. Therefore, larger, multicenter studies and broader patient populations, including outpatients, are warranted to confirm our findings.

## Conclusions

VMT scoring demonstrated excellent interobserver reproducibility and may serve as a practical screening or adjunctive tool for LVFP assessment using only B-mode echocardiography, pending further validation studies. It is considered to be valuable for physicians owing to its ease of use and adaptability, regardless of the level of clinical experience or the type of US system available. The use of VMT scoring has the potential to improve HF diagnosis and management in various clinical settings.
